# Effect of *Prunus cerasus *(Sour Cherry) on Nephrolithiasis in Children: The First Noninferiority Two-Arm Randomized Clinical Trial

**DOI:** 10.5152/tud.2024.23206

**Published:** 2024-03-01

**Authors:** Fatemeh Ghane-Sharbaf, Zahra Reza-Jafar, Elham Bakhtiari, Sara Saadat

**Affiliations:** 1 Department of Pediatrics, Mashhad University of Medical Sciences Faculty of Medicine, Mashhad, Iran; 2 Mashhad University of Medical Sciences, Faculty of Medicine, Mashhad, Iran; 3 Clinical Research Unit, Mashhad University of Medical Sciences, Mashhad, Iran

**Keywords:** *Prunus cerasus*, nephrolithiasis, child, Polycitra-K

## Abstract

**Objective:**

The present study evaluated the effect of *Prunus cerasus* (sour cherry) on children with nephrolithiasis.

**Methods:**

We conducted a randomized noninferiority controlled trial to evaluate the therapeutic efficacy of *P*.* cerasus* among children with nephrolithiasis. Subjects in the intervention group received 1.25 mL/kg of cherry concentrate once daily for 2 months, while the control group received 1 mL/kg Polycitra-K, which consists of 220 g citrate potassium and 68 g citric acid in 1000 mL sterile water. The major outcome was sonographically determined number and sizes of kidney stones, which were assessed before and after the trial.

**Results:**

Sixty-eight children completed the study. At trial onset, both groups were similar in baseline characteristics (*P* > .05). In within-group analysis, the number of stones significantly decreased in both groups (*P* < .05). After 2 months, the number of nephrolithiasis was 1.55 ± 0.49 and 1.47 ± 0.67 in the control and intervention groups, respectively (*P* value = .56). The percentage of change in calculi number was 44.11 ± 11.12 and 38.14 ± 14.08 in the control and intervention groups, respectively (*P* value = .08). At the end of the study, the urine pH was 6.46 ± 0.99 and 6.14 ± 0.83 in the control and intervention groups, respectively (*P* value = .19). Urine calcium and uric acid concentrations were 32.00 ± 12.32 and 28.95 ± 10.96 mg/mm (*P* value = .68) and 24.11 ± 10.58 and 30.03 ± 11.39 mg/mm (*P* value = .012) in control and intervention groups, respectively.

**Conclusion:**

Our clinical data supported the efficacy of sour cherry in the treatment of nephrolithiasis compared to Polycitra-K. Future randomized controlled trials are needed to confirm the present observation.

**Main Points**

- *Prunus cerasus *(sour cherry) is a common and highly used fruit worldwide which has a high content of citrate.
- Use of sour cherry for 2 months decreased the number and size of kidney stones in children.
- Sour cherry leads to a decrease in the urine uric acid during the intervention time.
- Sour cherry leads to an increase in the urine pH during the intervention time.
- Sour cherry is not inferior to poly-citrate potassium in the treatment of urolithiasis in children.

## Introduction

Nephrolithiasis is a common health problem with multifactorial etiologies. The exact prevalence of urolithiasis in children. is unknown. It has been estimated that children account for 2%-3% of stone formers worldwide.^1^ According to epidemiological studies, the prevalence of kidney stones is about 36 to 145 per 100 000 children.^1^ Several factors such as genetics, nutrition, and hydration status of hydration affect stone formation.^2^

Polycitra-K is commonly used to treat several types of nephrolithiasis because it forms a soluble calcium-citrate complex, thereby preventing the precipitation of calcium from urine as either calcium phosphate or calcium oxalate. Polycitra-K prevents the formation of uric acid and cystine stones by increasing urine pH.^3^ Alkali citrates are effective in the treatment of calcium oxalate, calcium phosphate, cystine, and uric acid stones. Citrates reduce urinary calcium via binding to calcium. This leads to the prevention of the crystallization of calcium oxalate and calcium phosphate stones.^4^ An old randomized clinical trial confirmed the efficacy of potassium citrate in calcium nephrolithiasis compared to placebo.^5^ Adherence of children to the prescribed Polycitra-K is generally challenging due to its bitter taste. In one Iranian study, only 62% of children treated with Polycitra-K showed adequate adherence.^6^

The popularity of natural products for the treatment of various illnesses appears to be rising. People believe that natural products are safer and have fewer side effects than synthetic drugs. Thus, there is a demand to study the effects of natural products with scientific rigor. Sour cherries (*Prunus cerasus*) are native to Europe and some parts of Asia. They are also grown in most parts of Iran. They are rich in vitamins A, C, and E, anthocyanins, and isoflavonoids.^7^ Pharmaceutical properties demonstrated for sour cherries include gastroprotective, anti-inflammatory, and antioxidant effects.^2,7-10^ Their consumption has been found to be beneficial for muscle soreness after exercise, gout, arterial hypertension, and cardiovascular diseases.^11,12^ Nevertheless, scientific evidence to support the clinical use of sour cherries is scarce. Animal experiments in rats suggest a therapeutic effect of *Cerasus avium* on calcium oxalate stones. For example, Azaryan et al. found that it reduced the number and sizes of calcium oxalate stones in rats.^13^

Sour cherries have a considerable content of citrate compared to other fruits.^14^ This led us to hypothesize that sour cherry may be effective in preventing or treating patients with nephrolithiasis. If so, the use of sour cherry extract could increase adherence due to its better palatability compared with Polycitra-K. However, we were unable to find a study demonstrating the efficacy of sour cherry compared with Polycitra-K in patients with nephrolithiasis. 

We therefore conducted the present randomized two-arm single-blind clinical study to evaluate the therapeutic efficacy of an oral sour cherry concentrate in children with nephrolithiasis in the form of a noninferiority trial with Polycitra-K treatment as the reference drug. 

## Material and Methods

**Figure 1.**
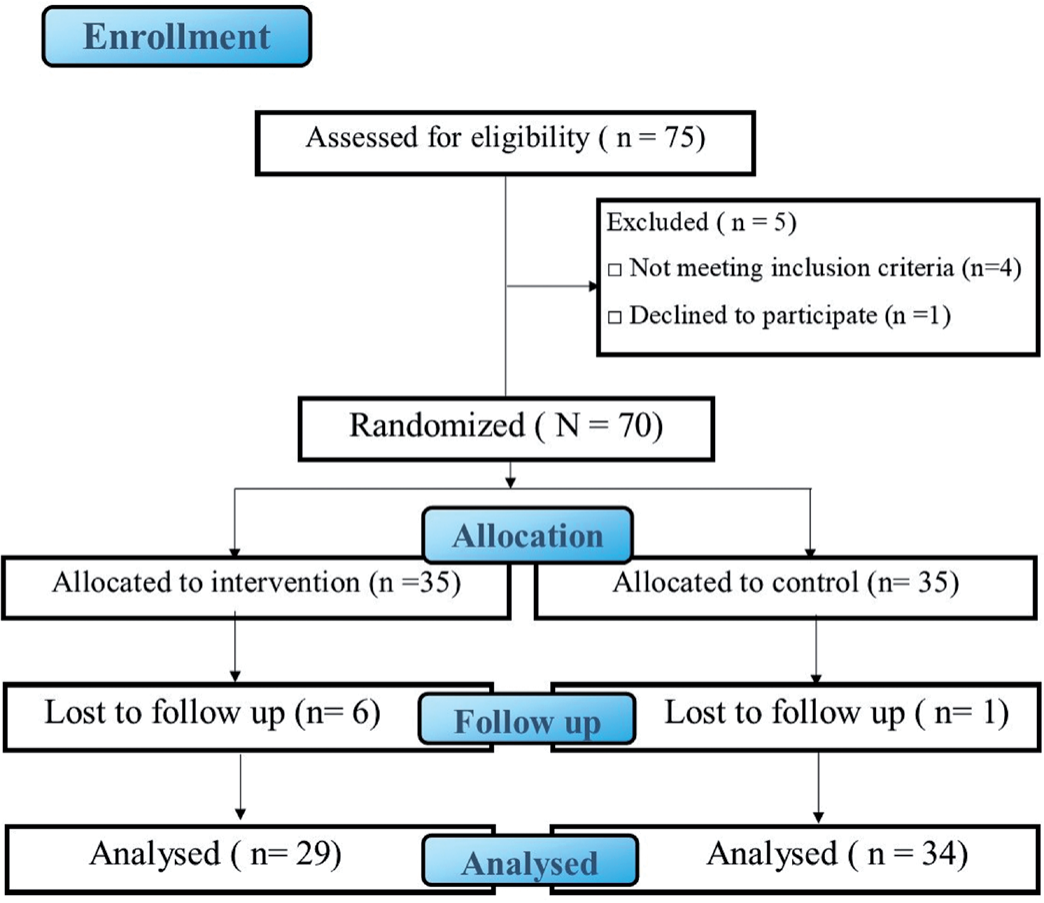
CONSORT diagram of the study.

The present study was a single-blind randomized clinical trial. It was conducted between 2020 and 2021 in a tertiary pediatric hospital, affiliated with the affiliated with the Mashhad University of Medical Sciences (MUMS), Mashhad, Iran. The study population comprised children with incident urolithiasis who had not been previously treated. The study was approved by the Medical Ethics Committee of the Mashhad University of Medical Sciences (Approval Number: IR.MUMS.fm.REC.1396.376; Date: September 27, 2017) and registered in the Iranian registry of clinical trials (IRCT) (Code: IRCT20170415033428N5, Date:June 29, 2020). Informedconsent was obtained from all subjects and/or their legal guardian(s). Seventy children aged 1-18 years were included. All clinical examinations were performed by a single pediatric nephrologist. Patients with urolithiasis and metabolic disorders including hyperuricosuria, hypercalciuria, hyperoxaluria, cystinuria, and hypocitraturia were included. Exclusion criteria were the presence of obstructive or infected calculi and lack of consent. A Consolidated Standards of Reporting Trials (CONSORT) flowchart is shown in Figure 1. The control treatment was Polycitra-K (Sepidaj Pharmaceutical Company). Controls recieved 1 mL/kg Polycitra-K, which consists of 220 g citrate potassium and 68 g citric acid in 1000 mL sterile water. One milliliter of Polycitra-K corresponds to 1 mmoL (2 mmEq) alkali. The intervention was sour cherry concentrate, which was purchased from Khoosheh Sorkhe Shargh Agro Industrial® (Tehran, Iran). The citrate content in the cherry concentrate was 8%, as determined by high-performance liquid chromatography (HPLC) (Testa Quality Control Laboratories, Mashhad, Iran). Cherry concentrate dosing in the intervention group was adjusted to match the amount of citrate consumed by control patients. Polycitra-K had a citrate content of 10%, while the cherry concentrate had 8% citrate. Therefore, the citrate content of 1 mL Polycitra-K was equivalent to 1.25 mL of cherry concentrate. 

Accordingly, the intervention group received 1.25 mL/kg of concentrate once daily for 2 months. All patients were instructed to follow a low-sodium diet and maintain high water intake. The primary outcome was the size and number of calculi determined by sonography (MyLab™X8 Doppler sonography machine, Esaote, Genova, Italy). An expert sonographer performed all ultrasound scans and was unaware of the patient groupings. Urine analysis and sonography of the kidneys and urinary tract were performed at the time of enrollment and 2 months after intervention for all patients. The following demographic and clinical data were recorded in a data collection sheet: sex, age at diagnosis, urine analysis (UA), and urine culture (UC) results, presence or absence hematuria (gross or microscopic), chief complaint (restless, dysuria, flank pain, vomiting, frequency, urinary tract infection (UTI), poor growth and change in voiding habits), abnormal clinical exam findings (flank mass, suprapubic pain, flank pain), family history of urolithiasis, blood test results (serum creatinine, uric acid, calcium, phosphorus, parathyroid hormone), and sonography findings. Urine pH was determined using dipstick chemistry. The urine sample was collected in the morning before breakfast. In toilet-training children, midstream urine was collected, while in non-toilet-training children, the sample was taken via a urine bag. All urine samples were sent to the laboratory within the first hour of sampling. 

Randomization was based on a commercial c**omputer-**generated random sequence. Allocation concealment was done via sealed envelopes. A person who was not on the research team did the patient's assignment. An expert who was not involved in the study evaluated primary outcomes. A 15% lesser response to treatment compared to the control group was considered as noninferior treatment. A sample size of 35 patients per group was considered appropriate for the initial study (a total of 70 patients).

### Statistical Analysis

Data were analyzed using SPSS version 16.0 (SPSS Inc.; Chicago, IL, USA). Quantitative data were presented as mean ± standard deviation (SD), and categorical data were presented as frequency and percentage. The association of qualitative variables was tested using Fisher's exact test or chi-square. Quantitative variables were compared using an independent-sample *t*-test. Within-group changes were assessed using paired *t*-tests. A *P*-value less than .05 was considered statistically significant.

## Results

### Baseline Characteristics

Among the 70 enrolled patients, 63 patients completed the study, with 34 in the control group and 29 in the intervention group. Seventeen patients (50%) and 14 patients (48.3%) were male in the control and intervention groups, respectively (*P* = .89). The mean age of patients in the control and intervention groups was 5.7 ± 2.31 and 5.29 ± 3.6 years respectively (*P* = .58). All patients were similar in baseline clinical and laboratory data (*P* > .05). Additionally, all patients had normal renal function at the time of enrollment (*P* > .05). Data are presented in Table 1 . 

### Number of Stones

The number of urolithiasis at the baseline was 2.79 ± 1.06 and 2.39 ± 1.05 in the control and intervention groups, respectively (*P* value = .22). After 2 months, the number of nephrolithiasis was 1.55 ± 0.49 and 1.47 ± 0.67 in the control and intervention groups, respectively (*P* value = .56). In within-group analysis, the number of stones significantly decreased in both groups (*P* < .05), but the percent of change in calculi number was 44.11 ± 11.12 and 38.14 ± 14.08 in the control and intervention groups, respectively (*P* value = .08, 95% CI: 1.75, 12.27).

### Urine pH

**Figure 2.**
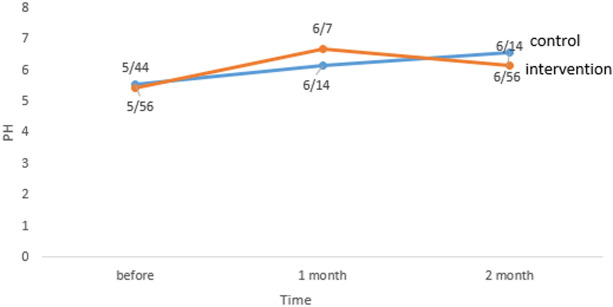
Changes in urine pH in groups over time.

The urine pH before the trial was 5.56 ± 0.27 and 5.44 ± 0.31 in the control and intervention groups, respectively (*P* value = .12). After 1 month, the urine pH was 6.14 ± 0.47 and 6.7 ± 0.3 in the control and intervention groups, respectively (*P* value = .57). After 2 months, the urine pH was 6.46 ± 0.99 and 6.14 ± 0.83 in the control and intervention groups, respectively (*P* value = .19). The data are presented in Table 2. The data are illustrated in Figure 2. 

### Urine Calcium

**Figure 3.**
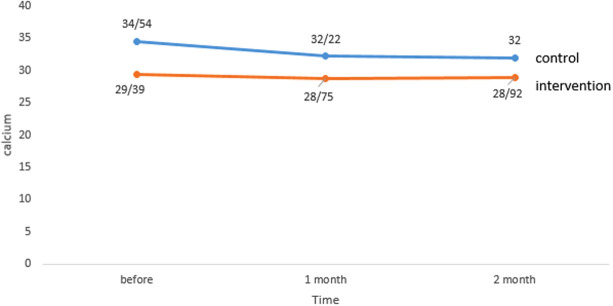
Changes in urine calcium in groups over time.

In a random sample, the urine calcium before the trial was 34.54 ± 29.32 and 29.39 ± 19.53 mg/mL) in the control and intervention groups, respectively (*P* value = .42). After 1 month, the urine calcium was 32.22 ± 23.06 and 28.75 ± 10.39 mg/mL in the control and intervention groups, respectively (*P* value = .45). After 2 months, the urine calcium was 32.00 ± 15.32 and 28.95 ± 18.96 mg/mL in the control and intervention groups, respectively (*P* value = .68). The data are presented in Table 2. The data are illustrated in Figure 3. 

### Urine Uric Acid

**Figure 4.**
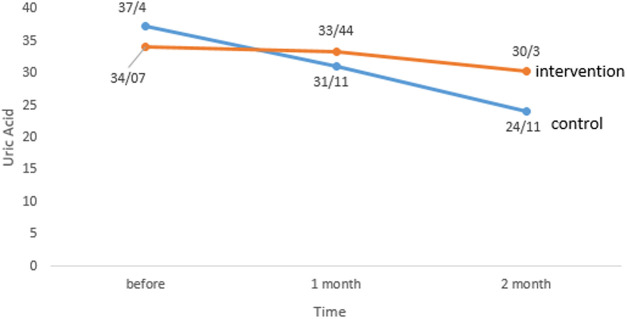
Changes in urine uric acid in groups over time.

In a random sample, the urine uric acid before the trial was 37.4 ± 24.77 and 34.07 ± 16.48 mg/mL in the control and intervention groups, respectively (*P* value = .54). After 1 month, the urine uric acid was 31.11 ± 24.95 and 33.44 ± 15.46 mg/mL in the control and intervention groups, respectively (*P* value = .66). After 2 months, the urine uric acid was 24.11 ± 14.58 and 30.03 ± 12.39 mg/mL in the control and intervention groups, respectively (*P* value=0.012). The data are presented in table 2. The data are illustrated in Figure 4. 

### Side Effects

There were no side effects associated with the intervention. However, in the control group, 2 patients (5.88%) had mild to moderate diarrhea.

## Discussion

In a randomized two-arm single-blind clinical study, 68 children with urolithiasis were studied evaluating the effect of sour cherry concentrate on the number of nephrolithiasis compared to Polycitra-K. Results showed that sour cherry concentrate is not inferior to Polycitra-K in the treatment of nephrolithiasis. Despite some evidence regarding the traditional use of *P. cerasus* in the management of kidney stones,^15^ this study is the first clinical trial supporting the beneficial effect of sour cherry concentrate in the treatment of nephrolithiasis. Our results are particularly important in children because the compliance of Polycitra-K in children is rather low. Certain pharmacological properties of *P. cerasus* were studied previously.^16-18^ However, the current study demonstrates its clinical efficacy for the treatment of nephrolithiasis for the first time. Many medicinal plants have been used to treat urolithiasis in animal and clinical experiments^1,2^ with variable results.^15,19^ According to Shirani, *Arnebia euchroma* root extract efficacy dissolved calcium oxalate and calcium phosphate stones in vitro.^20^ In a meta-analysis of clinical trials, it has been shown that Cystone®(ayurvedic proprietary medicine) decreased the size and number of kidney stones compared to placebo.^19^ In another systematic review, the authors concluded that there is insufficient data to support the use of herbal products for the treatment of urolithiasis.^21^ Singh et al conducted a randomized clinical trial comparing the efficacy of another ayurvedic plant extract, Calcury®, with potassium citrate in stone-forming patients and concluded that phytotherapy effectively reduces stone size and improves biochemical parameters, such as hypercalcemia, oxaluria, and urinary citrate and pH.^22^

The beneficial effect of *Prunus mahaleb* extract on kidney stone formation was studied in BALB/c mice. In this study, 500 mg of *P. mahaleb* effectively prevented the formation of kidney stones and improved serum creatinine and urea levels compared to the control group without adverse effects.^23^ Azaryan reported that 200 and 400 mg/kg aqueous extract of *C. avium* stem powder have a therapeutic effect on calcium oxalate stones in rats with nephrolithiasis and reduce the number of calcium oxalate deposits.^13^ Mehmet et al reported that patients with nephrolithiasis consumed less cherry (as a fruit) compared to healthy controls.^2^ In another clinical study on healthy volunteers, it was reported that the capsules of *Prunus avium* (cherry) stalk increase the volume of urine and that it could be used as a mild diuretic without identified side effects.^24^ In our study, *P. cerasus* was as effective as Polycitra-K in reducing the stone load. Despite this, the measured urine calcium and uric acid excretion did not decrease as in the control group. Polycitra-K is considered the first-line treatment in metabolic nephrolithiasis. It alkalinizes the urine which leads to the dissolution of calcium, uric acid, and other crystals.^3^ According to our results, *P. cerasus* also raises the urine pH which is clinically valuable, but less than potassium citrate. 

Our clinical data support the hypothesis that sour cherry concentrate is not inferior to the standard treatment of nephrolithiasis with Polycitra-K. Sour cherry concentrate led to a 38% decrease in the number of calculi, which was similar to the treatment effect of Polycitra-K. Additional clinical studies are warranted, in children and adults, to confirm the efficacy and the benefit of the described treatment. 

## Limitations

Because of the distinctive taste and appearance of sour cherry concentrate, it was not possible to blind the patients. The follow-up time in this study was rather short, which may have affected the results. Longer durations of treatment and follow-up are suggested in future studies.

## Availability of data and material:

All data generated or analyzed during this study are available from the corresponding author on a reasonable request.

## Figures and Tables

**Table 1.** Demographic, Clinical, and Laboratory Characteristics of Patients with Urolithiasis

**Variables**	**Control Group** **(n = 34)**	**Intervention Group** **(n = 29)**	***P***
Sex (male)	17 (50%)	14 (48.3%)	.89*
Restless	8 (23.5%)	6 (20.68%)	.78*
Dysuria	6 (17.6%)	7 (24.1%)	.52*
Abdominal pain	12 (35.3%)	10 (34.5%)	.94*
Suprapubic pain	7 (20.6%)	4 (13.8%)	.47*
History of UTI	4 (11.8%)	2 (6.9%)	.51*
History of urolithiasis	7 (20.6%)	4 (13.8%)	.60*
Urine retention	1 (2.9%)	0	.35*
Gross hematuria	3 (8.8%)	1 (3.4%)	.38*
Positive urine culture	5 (14.7%)	3 (10.3%)	.60*
Nitroprusside test	3 (10%)	0	.11*
Nephrocalcinosis	2 (5.9%)	0	.18*
Microscopic hematuria	9 (26.5%)	3 (10.3%)	.10*
Urine color change	5 (14.7%)	2 (6.9%)	.32*
Stone passing presentation	2 (5.9%)	2 (6.9%)	.86*
WBC in urine^#^	12 (41.4%)	14 (48.3%)	.59*
RBC in urine^#^	15 (51.7%)	10 (34.5%)	.18*
Urine crystal	11 (32.4%)	10 (34.5%)	.88*
Serum level of BUN (mg/dL)	17.5 ± 7.1	16.66 ± 5.2	.64+
Serum level of calcium (mg/dL)	9.55 ± 0.55	9.74 ± 0.36	.13+
Serum level of creatinine (mg/dL)	0.43 ± 0.17	0.39 ± 0.12	.39+
Calcium/creatinine (urine)	0.48 ± 0.38	0.41 ± 0.14	.35+
Uric acid/creatinine (urine)	1.56 ± 1.24	1.6 ± 0.35	.86+
Urine pH	5.56 ± 0.27	5.44 ± 0.31	.11+

RBC, red blood cell; UTI, urinary tract infection; WBC, white blood cell.

^#^More than 5 cells in high power field.

*Chi-square or Fisher's exact test.

^+^Independent *t-*test.

**Table 2.** The urine pH, Calcium, and Uric Acid in Children with Nephrolithiasis Before and After the Study

Variables	Groups	Before the Study	After 1 Month	After 2 Months
Urine pH	Control	5.56 ± 0.27	6.14 ± 0.47	6.46 ± 0.99
	Intervention	5.44 ± 0.31	6.7 ± 0.3	6.14 ± 0.83
	*P* value*	.12	.57	.19
Urine calcium (mg/mL)	Control	34.54 ± 29.32	32.22 ± 23.06	32.00 ± 15.32
	Intervention	29.39 ± 19.53	28.75 ± 10.39	28.95 ± 18.96
	*P* value*	.42	.45	.68
Urine acid uric (mg/mL)	Control	37.4 ± 24.77	31.11 ± 24.95	24.11 ± 14.58
	Intervention	34.07 ± 16.48	33.44 ± 15.46	30.03 ± 12.39
	*P* value*	.54	.66	.012

*Repeated measure ANOVA.

## References

[1] Chu DI Tasian GE Copelovitch L . Pediatric kidney stones—avoidance and treatment. Curr Treat Options Pediatr. 2016;2(2):104 111. (10.1007/s40746-016-0046-8) 27766194 PMC5067072

[2] Icer MA Gezmen-Karadag M . The potential effects of dietary food and beverage intakes on the risk of kidney stone formation. Rev Nutr. 2019;32. (10.1590/1678-9865201932e190029)

[3] Carvalho M Erbano BO Kuwaki EY , et al. Effect of potassium citrate supplement on stone recurrence before or after lithotripsy: systematic review and meta-analysis. Urolithiasis. 2017;45(5):449 455. (10.1007/s00240-016-0950-1) 27915395

[4] Fuselier HA Ward DM Lindberg JS , et al. Urinary tamm-Horsfall protein increased after potassium citrate therapy in calcium stone formers. Urology. 1995;45(6):942 946. (10.1016/s0090-4295(99)80112-5) 7771027

[5] Barcelo P Wuhl O Servitge E Rousaud A Pak CY . Randomized double-blind study of potassium citrate in idiopathic hypocitraturic calcium nephrolithiasis. J Urol. 1993;150(6):1761 1764. (10.1016/s0022-5347(17)35888-3) 8230497

[6] Sorkhi H Saeedizand N Poornasrollah M Bijani A Shafi H . Efficacy of potassium polycitrate on renal stone and microlithiasis predisposed by metabolic disorders. Caspian J Intern Med. 2017;8(4):296 300. (10.22088/cjim.8.4.296) 29201321 PMC5686309

[7] Raafat K El-Darra N Saleh FA . Gastroprotective and anti-inflammatory effects of Prunus cerasus phytochemicals and their possible mechanisms of action. J Trad Complement Med. 2020;10(4):345 353. (10.1016/j.jtcme.2019.06.001) PMC7365781 32695651

[8] Varga B Priksz D Lampé N , et al. Protective effect of prunus cerasus (sour cherry) seed extract on the recovery of ischemia/reperfusion-induced retinal damage in Zucker diabetic fatty rat. Molecules. 2017;22(10):1782. (10.3390/molecules22101782) 29065463 PMC6151469

[9] Tall JM Seeram NP Zhao C Nair MG Meyer RA Raja SN . Tart cherry anthocyanins suppress inflammation-induced pain behavior in rat. Behav Brain Res. 2004;153(1):181 188. (10.1016/j.bbr.2003.11.011) 15219719

[10] Blando F Gerardi C Nicoletti I . Sour cherry (Prunus cerasus L) anthocyanins as ingredients for functional foods. J Biomed Biotechnol. 2004;2004(5):253 258. (10.1155/S1110724304404136) 15577186 PMC1082898

[11] Kelley DS Adkins Y Laugero KD . A review of the health benefits of cherries. Nutrients. 2018;10(3):368. (10.3390/nu10030368) 29562604 PMC5872786

[12] Collins MW Saag KG Singh JA . Is there a role for cherries in the management of gout? Ther Adv Musculoskelet Dis. 2019;11:1759720X19847018. (10.1177/1759720X19847018) PMC6535740 31205513

[13] Azaryan E Malekaneh M Shemshadi Nejad MS Haghighi F . Therapeutic effects of aqueous extracts of Cerasus avium stem on ethylene glycol-induced kidney calculi in rats. Urol J. 2017;14(4):4024 4029. 28670670

[14] Karaat FE Gündüz K Saraçoǧlu O Yıldırım H . Pomological and phytochemical evaluation of different cherry species: mahaleb (Prunus mahaleb L.), wild sweet cherry (Prunus avium L.) and wild sour cherry (Prunus cerasus L.), sweet and sour cherry cultivars. asphc. 2019;18(4):181 191. (10.24326/asphc.2019.4.17)

[15] Bahmani M Baharvand-Ahmadi B Tajeddini P Rafieian-Kopaei M Naghdi N . Identification of medicinal plants for the treatment of kidney and urinary stones. J Ren Inj Prev. 2016;5(3):129 133. (10.15171/jrip.2016.27) 27689108 PMC5039998

[16] Berroukche A Benreguieg M Terras M , et al. Antibacterial effects of Prunus cerasus and Chamaemelum nobile against drug resistant strains induced urinary disorders. East Afr Scholars J Med Sci. 2018;1(2):26 31.

[17] Li R Tan Y Li Y , et al. Effects of tart cherry powder on serum uric acid in hyperuricemia rat model. Evid Based Complement Alternat Med. 2020;2020:1454305. (10.1155/2020/1454305) 32774405 PMC7396008

[18] Martinelli I Tomassoni D Bellitto V , et al. Anti-inflammatory and antioxidant properties of tart cherry consumption in the heart of obese rats. Biology. 2022;11(5):646. (10.3390/biology11050646) 35625374 PMC9138407

[19] Azarfar A Rafiee Z Ravanshad Y Saber Moghadam NS Bakhtiari E . Effect of herbal formulation "cystone®" on urolithiasis. Jundishapur J Nat Pharm Prod. 2020;15(3). (10.5812/jjnpp.69246)

[20] Shirani M Arjaki D Kheiri S Bijad E Mohammadi S Lorigooini Z . An in vitro screening potential traditional medicinal plants for nephrolithiasis. Clin Phytosci. 2020;6:1 8.

[21] Khan A Khan SR . Clinical studies of medicinal plants for their antiurolithic effects: A systematic review. Longhua Chin Med. 2022;5:16 16. (10.21037/lcm-21-51)

[22] Singh I Bishnoi I Agarwal V Bhatt S . Prospective randomized clinical trial comparing phytotherapy with potassium citrate in management of minimal burden (≤ 8 mm) nephrolithiasis. Urol Ann. 2011;3(2):75 81. (10.4103/0974-7796.82172) 21747596 PMC3130482

[23] Azadbakht M Dashti A Vahedi L . Effect of Prunus mahaleb L. seed extract on ethylene glycol-and ammonium chloride-induced urolithiasis in BALB/c mice. Iran J Med Sci. 2020;45(2):134 139. 32210490 10.30476/IJMS.2019.45774 PMC7071555

[24] Hooman N Mojab F Nickavar B Pouryousefi-Kermani P . Diuretic effect of powdered Cerasus avium (cherry) tails on healthy volunteers. Pak J Pharm Sci. 2009;22(4):381 383. 19783515

